# Research on the impact of pilot free trade zones on urban green development: A case study based on the Yangtze River Economic Belt in China

**DOI:** 10.1371/journal.pone.0303626

**Published:** 2024-05-24

**Authors:** Weiwei Wu, Xiaoyong Tian, Yating Liu, Weitong Huang

**Affiliations:** Hunan Institute of Science and Technology, College of Economics and Management, Yueyang, Hunan, China; East China Normal University, CHINA

## Abstract

Green development is an important component of China’s new development concept. Pilot Free Trade Zones (PFTZs), as “experimental fields” for promoting reform, deepening opening-up, and raising the level of an open economy, are important open areas for China to promote green development. However, existing related research is not extensive. This article takes PFTZs as quasi-natural experiments, with the Yangtze River Economic Belt (YREB) as the research area. Based on urban panel data from 2006 to 2020, using multi-period differences-in-differences and spatial differences-in-differences models, it explores the impact effects of PFTZs on urban green development and their potential mechanisms. The research findings indicate: (1) Overall, PFTZs significantly promote urban green development, with variations in impact effects due to different batches and locations of establishment. (2) Mechanism tests show that PFTZs mainly promote urban green development by stimulating technological innovation, industrial upgrading, and reducing government intervention. (3) From the perspective of spatial spillover effects, the establishment of PFTZs not only promotes the green development process in the host cities but also has a promoting effect on the green development of surrounding cities.

## 1. Introduction

Since the reform and opening-up, China has experienced a period of rapid economic growth. However, the environmental pollution caused by the traditional extensive development model of high input, high consumption, and low output has become increasingly severe, threatening the sustainable development of cities. It is urgent to reduce pollution and emissions to ensure a virtuous ecological cycle. According to the “China’s Ecological Environment Status Bulletin 2021” (http://www.mee.gov.cn/), 121 cities out of 339 prefecture-level cities and above in China exceeded the environmental control quality standards, accounting for 35.7%. The report of the 20th National Congress of the Communist Party of China proposed that high-quality development is the primary task for building a socialist modern country in an all-round way, and promoting green development in economy and society is the key to achieving high-quality development. Enhancing green development is an important grip to promote high-quality economic development and sustainability. During the critical period of China’s economic transition to high-quality development, green economic transformation is an important guarantee for maintaining long-term and stable development by improving the quality and quantity of China’s economy. It is also a wise move to meet people’s needs for abundant material resources while enjoying a high-quality environment with green water and green mountains [1,2]. “Green development” is a modern sustainable development model aimed at efficiency, harmony, and sustainability, which is embodied through various ways of strengthening ecological and environmental protection, such as efficient use of resources, improved efficiency of clean production, and enhanced end-of-pipe treatment based on the traditional extensive development model [3].

To comply with the trend of economic globalization and expand openness, the Chinese government has actively promoted the construction of PFTZs to foster a new pattern of comprehensive openness [4]. PFTZs, serving as a policy effect to promote the free flow of domestic and foreign factor resources, efficient allocation of resources, and deep integration with global markets, offer solutions to environmental pollution from the perspective of institutional innovation.

In 2021, the Ministry of Ecology and Environment of China issued the “Guiding Opinions on Strengthening Ecological and Environmental Protection in Pilot Free Trade Zones and Promoting High-Quality Development” (Circular on Comprehensive Environmental Issues〔2021〕 No. 44) (http://www.mee.gov.cn/). The document emphasized the need to focus on promoting high-quality development and deepening supply-side structural reform, intensify the battle against pollution, innovate ecological and environmental management models and systems, comprehensively improve the level of ecological and environmental protection in PFTZs, and promote harmonious development between trade, investment, and the ecological environment. The concept of green development should be integrated throughout the entire process of PFTZ construction, fostering advanced green manufacturing, green services, green trade, and green supply chains.

Since the issuance of the policy, PFTZs across the country have responded actively. Shanghai PFTZ, for example, has incorporated carbon peak and carbon neutrality into its overall economic and social development plan, established and improved incentive and restraint mechanisms for reducing pollution emissions and greenhouse gas emissions, and promoted a comprehensive green transformation of social development. The Zhejiang PFTZ has implemented intelligent upgrades to its logistics information system within the free trade zone, introducing green management measures for express logistics packaging [5]. Since the issuance of relevant policies, there is an urgent need to verify the substantial effects of PFTZ construction on urban green development.

The YREB spans the eastern, central, and western regions of China, passing through nine provinces and two municipalities. Due to its diverse resource endowments and unique ecological characteristics, it has become an experimental highland for promoting China’s economic transformation, necessitating the integration of green development concepts throughout its construction process. In 2022, the gross domestic product of the YREB reached 55.98 trillion yuan, an increase of 3.0% year-on-year, with both population and per capita GDP exceeding 40% of the national average [6]. Since the establishment of the China (Shanghai) PFTZ in 2013, China has set up 21 PFTZs, nine of which are located within the YREB, covering 23 cities (see Table 1). More than one-fifth of these cities have established PFTZs. Therefore, studying the environmental effects of the implementation of pilot policies in the PFTZs along the YREB is crucial for better leveraging the strategic fulcrum role of orderly expansion and strengthening the connotation of these zones. However, most existing policy evaluation studies have focused on exploring the economic effects of coastal PFTZs [7,8]. Whether the PFTZs along the YREB can replicate the institutional innovation achievements of coastal PFTZs remains a subject for further research.

**Table 1:** Planning of PFTZs within the YREB.

PFTZs	Relying on Urban Area	Scale (KM^2^)
Shanghai PFTZ	Shanghai	120.72
Zhejiang PFTZ	Zhoushan, Ningbo, Hangzhou, Jinhua, Zhejiang	119.95
Hubei PFTZ	Wuhan, Xiangyang, Yichang	119.96
Chongqing PFTZ	Chongqing	119.98
Sichuan PFTZ	Nanjing, Suzhou, Lianyungang	119.99
Jiangsu PFTZ	Kunming, Dehong, Honghe	119.97
Yunnan PFTZ	Kunming, Dehong, Honghe	119.86
Hunan PFTZ	Changsha, Yueyang, Chenzhou	119.76
Anhui PFTZ	Hefei, Wuhu, Bengbu	119.86

Given this background, this paper takes cities where PFTZs are located as the research subjects and aims to explore the driving effects of PFTZ construction in the YREB on urban green development from both theoretical and empirical perspectives. Compared to existing literature, the potential marginal contributions of this paper lie in the following aspects: Firstly, in terms of research perspective, while existing studies have focused on the influencing factors of urban green development, this paper expands the analysis by emphasizing the perspective of PFTZs construction. Secondly, in terms of research content, this paper not only analyzes the practical effects of PFTZs construction on promoting urban green development from both theoretical and empirical levels, but also strives to reveal the mechanism underlying these effects. Finally, in terms of measurement methods and data usage, this paper employs a spatial double difference model to explore the spatial effects of PFTZs construction. Distinguishing from previous studies using provincial-level panel data, this paper further narrows down the research subjects to the level of PFTZs areas within the YREB and utilizes city-level data to enhance the applicability of the conclusions.

The study’s contributions in comparison to existing research are outlined as follows: Firstly, while previous literature has extensively addressed the economic impacts of PFTZs construction, this paper focuses on the influence of PFTZs construction on urban green development, a topic that has received limited attention. Thus, this study utilizes panel data from 99 major cities in the YREB to investigate the impact of PFTZs construction on urban green development, aiming to fill this research gap. Secondly, the study delves into the mediating effects of technological innovation, industrial upgrading, and government intervention to offer insights for refining and promoting PFTZs policies. Furthermore, by analyzing the establishment batches of PFTZs and the geographic locations of cities, the study examines the heterogeneous impact of PFTZs construction on urban green development, providing valuable guidance for promoting green development. Lastly, while previous studies have only considered the impact of PFTZs construction on the host city, this paper incorporates the spatial double difference method to explore the spatial spillover effect of PFTZs construction on green development from a spatial perspective, thereby enriching the research landscape.

The paper’s remaining structure is as follows: section 2 examines the pertinent literature, section 3 outlines the research hypotheses, section 4 details the research design, section 5 analyzes the empirical findings, section 6 discusses further research, and section 7 conclusions and research recommendations.

## 2. Literature review

### 2.1 Urban green development

Urban green development places strong emphasis on the integrated consideration of economic, trade, and environmental factors, with a focus on ensuring that all economic activities support ecology and the green economy [9]. The Green Total Factor Productivity (GTFP) is widely recognized as a key tool for measuring the level of regional green development [10–12]. GTFP is designed to assess and improve both economic and environmental performance, making it an important method for evaluating green development [13]. Researchers have primarily focused on two main areas of study: indicator measurement and influencing factors. Indicator measurement is mainly achieved through two methods, including Stochastic Frontier Analysis (SFA) [14] and DEA [15,16]. Additionally, the Global Malmquist-Luenberger (GML) productivity index method has been found to be more effective than other indices in reflecting the dynamics of GTFP efficiency values over time [17]. Therefore, this paper adopts the relaxation super-efficiency measurement model combined with the GML index to calculate GTFP. In terms of influencing factors, most scholars concur that environmental regulations have a long-term positive impact on China’s marine economy and that these regulations have a single-threshold effect on GTFP [18]. Low-carbon policies rely on technological progress to promote green productivity in pilot cities and their neighboring areas [19]. Human capital [20], foreign direct investment [21], and the digital economy [22] contribute to GTFP growth by fostering green innovation and entrepreneurial activities. These studies provide valuable insights and governance experience for regional green development.

### 2.2 PFTZ policy impact on urban green development

PFTZs represent a unique form of economic zones focused on institutional innovation with the goal of advancing international trade, investment, and economic growth through the relaxation of trade and investment constraints. Numerous studies have been conducted on the economic policy implications of PFTZs, primarily concentrating on economic growth [23,24], foreign direct investment [25], import and export trade [26,27], industrial structure [28], and technological innovation [29]. The findings of various scholars’ research are largely consistent, indicating significant positive economic impacts of PFTZs. However, it is crucial for resource-rich regions like PFTZs to prioritize the development and implementation of low-carbon environmental measures [30]. While some studies have demonstrated a clear causal relationship between PFTZs construction and environmental quality [31], there remains a lack of consensus on the association between PFTZs and environmental quality. Some scholars argue that the establishment of PFTZs could exacerbate the environmental burden and threaten the environment, while others contend that trade liberalization could lead to developing countries becoming “pollution havens” for developed nations [32]. Additionally, there are concerns that trade openness may drive economic expansion at the cost of increased energy consumption and carbon dioxide emissions [33].

Some scholars, however, propose a contradictory viewpoint, contending that the creation of PFTZs is advantageous for sustainable development and could enhance environmental quality. According to Erdogan [34], trade liberalization can effectively mitigate the conflict between demand and resources and promote technological advancement, thereby enhancing environmental quality. Additionally, Ramli and Munisamy [35] discovered that countries with PFTZs demonstrate significantly higher eco-efficiency compared to those without, potentially due to increased technological spillovers and innovations from foreign trade. Akbari et al. [36] argued that PFTZs can effectively integrate domestic production factors with international advanced technologies, driving eco-innovation and environmental protection through improved research and development of green technologies. Empirical studies by Jiang et al. [37] and Ma et al. [38] on the Shanghai PFTZ revealed significant institutional dividends and a positive correlation between PFTZs and GTFP, albeit with diminishing effects over time. Furthermore, Chang et al. [2] found that the establishment of PFTZs significantly fosters regional green and high-quality development, while Zhang and Zhou [8] identified that PFTZs enhance regional green total factor energy efficiency through industrial structure upgrades and promotion of green technological innovation.

Based on the aforementioned literature, prior research has predominantly concentrated on the economic ramifications of establishing PFTZs. With the continual implementation of the concept of green sustainable development, an increasing number of scholars are prioritizing the notion that economic advancement should not come at the expense of the environment. The symbiosis between economic growth and environmental sustainability needs to be underscored [39]. Nevertheless, there are evident deficiencies in the existing research. The majority of the literature on PFTZs and green development utilizes provincial data or a specific PFTZ as the subject for empirical analysis. China has currently established 21 PFTZs with extensive regional coverage, and empirical analyses based on provincial data or individual PFTZs have limitations that may exacerbate or obscure actual impacts, consequently resulting in the biased nature of relevant measures. Additionally, a substantial number of studies have verified that the establishment of PFTZs has a discernible influence on urban green development from an environmental protection perspective, yet a consensus has not been reached. Scholars have also seldom delved into whether the influence of PFTZs establishment on the green development of their respective regions will affect the green development of surrounding neighboring cities simultaneously. Therefore, this study selects 99 prefecture-level cities in China’s YREB as its research sample to investigate the influence of PFTZs establishment on cities’ green development, aiming to provide theoretical and empirical guidance for the further construction of PFTZs in China.

## 3. Theoretical mechanisms and research hypotheses

### 3.1 Mechanisms of the impact of PFTZs on urban green development

**Fig 1:**
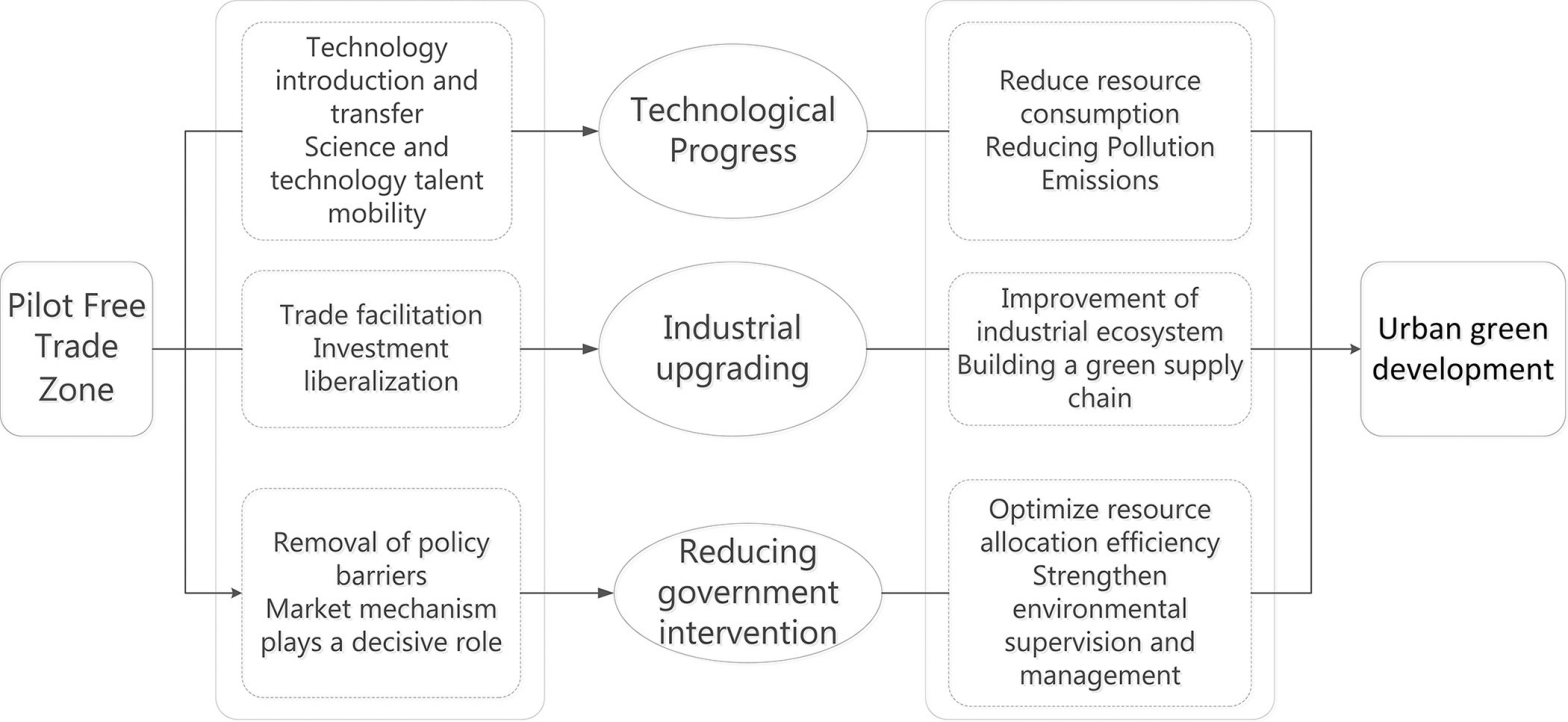
Mechanistic Pathways for PFTZs to Influence Urban Green Development.

PFTZs, as the vanguards of trade liberalization and policy innovation, possess substantial autonomy and favorable policies, offering fresh momentum to urban green development. As depicted in Fig 1, PFTZs have the capacity to enhance urban green development through technological advancements, industrial modernization, and diminished government involvement.

The establishment of PFTZs attracts both domestic and international scientific and technological talents and experts, fostering the exchange and collaboration of technological innovation resources. This in turn, catalyzes new innovative ideas, technological solutions, and business models, and promotes the transformation and application of scientific and technological achievements [40]. The series of policy measures formulated within PFTZs draw in a significant number of international enterprises, bringing advanced technology and management experience [41], thus propelling the technological upgrading and innovation of local enterprises. The concentration of funds and resources elevates the technological prowess of local enterprises. Moreover, PFTZs play a crucial role in promoting technological progress, enhancing the level of environmental protection in cities, and alleviating resource consumption and pollution emissions, thereby providing essential support and impetus for urban green development [29,42].

The establishment of PFTZs typically facilitates the enhancement and streamlining of the industrial chain [7], fosters the integration and collaboration of diverse industries [8], establishes a more comprehensive and efficient industrial ecosystem, minimizes resource wastage, enhances resource utilization efficiency, and decreases energy consumption and emissions [18], thus advancing the eco-friendly development of the city. PFTZs create a conducive environment and circumstances for the growth of emerging industries, spur the establishment and advancement of green supply chains [3,43], and drive the urban transition towards sustainability. Consequently, by driving industrial upgrading and transformation, PFTZs enhance the industrial structure and technological prowess of cities [41], diminish resource consumption and environmental contamination, and propel cities towards a more sustainable and eco-friendly trajectory.

The establishment of PFTZs are often accompanied by market-oriented reforms aimed at reducing government intervention in the market and empowering the market to significantly influence resource allocation. This optimization of resource allocation stimulates innovative activities within enterprises and fosters the shift towards green technologies and industries, thereby promoting the green development of cities [25]. However, it’s important to note that reducing government intervention does not equate to relaxation of environmental regulation. On the contrary, PFTZs typically enhance environmental quality by reinforcing environmental regulation and enforcement [44], granting enterprises more autonomy in selecting environmental technologies and green production methods. This flexibility enables enterprises to make environmentally mindful investments and adopt eco-friendly production methods, ultimately reducing environmental pollution and propelling green urban development. Moreover, the reduction of government intervention facilitates resource optimization, decreases resource wastage, and promotes the efficient utilization of resources [45]. Consequently, PFTZs indirectly facilitate the progression of urban green development by minimizing government intervention, boosting market mechanisms, increasing innovative activities and environmental protection investments, optimizing resource allocation, and strengthening environmental regulation.

Based on the above analysis, this paper puts forward the following research hypotheses.

Hypothesis 1 (H1): The construction of PFTZs play a crucial role in promoting urban green development.
Hypothesis 2a (H2a): PFTZs can facilitate urban green development through technological advancement.
Hypothesis 2b (H2b): PFTZs can stimulate urban green development through industrial upgrading.
Hypothesis 2c (H2c): PFTZs can encourage urban green development by reducing government intervention.

### 3.2 Spatial effects of PFTZs on urban green development

PFTZs not only represent a development in their own right, but also serve as a model for other regions to emulate. This forms a replicable and scalable model that allows other regions to benefit from the experiences of PFTZs in achieving high-quality development, creating a win-win situation for both the economy and the environment. Economic and trade exchanges between cities create spatial interconnections [46]. Policies oriented towards specific locations often lead to the reallocation of regional resources [47] It has been observed that the establishment of PFTZs can have both radiation and siphoning effects on the economic development of neighboring cities. Therefore, the third question explored in this paper is the potential spatial spillover effect of the establishment of PFTZs on urban green development as well. On the one hand, in the face of significant environmental pressure and the dual evaluation of economic and environmental performance, local governments have consistently implemented policies favorable to urban green development. As a result, the effective policy measures of PFTZs on urban green development have a demonstrative effect on neighboring cities [48]. On the other hand, PFTZs create a favorable market environment for market players through institutional reforms, exerting a strong siphoning effect on high-level factors such as talents and high-technology industries in neighboring regions. The clustering of high-level factors will inevitably promote the upgrade of the local industrial structure, potentially leading to the elimination of high-energy, high-emission, and high-pollution industries or their relocation [49]. This may hinder the green development of neighboring cities. Therefore, the spillover effect of the establishment of PFTZs on the green development of neighboring cities depends on the absolute value of the demonstration effect and the siphoning effect. Based on the above analysis, the following research hypotheses are proposed:

Hypothesis 3 (H3): The creation of PFTZs are expected to generate spatial spillover effects on the sustainable development of adjacent cities.

## 4. Study design

### 4.1 Benchmark model

The PFTZ policy is established by the state in batches, which can be regarded as a “quasi-natural experiment”. This paper draws on Beck [50] to construct a panel two-way fixed-effects model to estimate the impact of PFTZs policies on urban green development in the YREB. The specific model is as follows:

$$\begin{array}{l} GTFP_{it}=\alpha_{0}+\alpha_{1}PFTZ_{it}+\alpha_{2}Control_{it}+\varphi_{t}+\delta_{i}+\varepsilon_{it}\\ PFTZ_{it}=Treat_{i}\times Post_{t} \end{array}$$ (1)

where, *GTFP*_*it*_ denotes GTFP by province; the coefficient *α*_1_ is the net effect of the PFTZ on the GTFP of cities, and a positive value and a larger value indicates a larger positive effect; *Control*_*it*_ denotes control variables, *α*_2_ denotes coefficients of control variables, *φ*_*t*_ denotes time fixed effects; *δ*_*i*_ denotes individual fixed effects; and *ε*_*it*_ denotes random error terms.

### 4.2 Data sources

Considering the scientific and rational nature of the data, this paper selects the panel data of cities in the YERB from 2009 to 2020 for empirical analysis. Bijie and Tongren cities are excluded due to serious data deficiencies. In addition, nine cities, namely Hangzhou, Ningbo, Jinhua, Hefei, Wuhu, Bengbu, Changsha, Yueyang and Chenzhou, are excluded due to the establishment of a PFTZ in September 2020, where the institutional dividends are not fully released, and the impact on the experimental results is not avoided. Finally, 99 cities in the YREB were selected as the study sample, and the measured data were mainly obtained from the China City Statistical Yearbook, the China Environmental Statistical Yearbook, and the statistical yearbooks and statistical bulletins of provinces and cities.

### 4.3 Variable interpretation

#### 4.3.1 Explained variable

**GTFP** In this paper, we calculate the GTFP using a measurement-unexpected model based on super-efficient relaxation. It is assumed that there are *n* decision-making units production system, decision-making units are composed of inputs, desired outputs and non-desired outputs input-output vectors, and input *m* units get *S*_1_ desired outputs and *S*_2_ non-desired outputs. Based on the set of production possibilities, the green total factor productivity of region *m* in period *i* is established as an explanatory variable:

$$\begin{array}{l} \rho *=\min\frac{\frac{1}{m}\sum_{i=1}^{m}\frac{\bar{x_{i}}}{x_{io}}}{\frac{1}{S_{1}+S_{2}}(\sum_{r=1}^{S_{1}}\frac{\bar{y_{i}^{g}}}{y_{r0}^{g}}+\sum_{r=1}^{S_{2}}\frac{\bar{y_{r}^{b}}}{y_{r0}^{b}})},s.t.\left\{ \begin{array}{c} \bar{x}\geq \sum_{j=1,\neq k}^{n}\theta_{j}x_{j}\\ \bar{y^{g}}\leq \sum_{j=1,\neq k}^{n}\theta_{j}y_{j}^{g}\\ \bar{y^{b}}\geq \sum_{j=1,\neq k}^{n}\theta_{j}y_{j}^{b}\\ \begin{array}{l} \bar{x}\geq x_{0},\bar{y^{g}}\geq y_{o}^{g},\bar{y^{b}}\geq y_{o}^{b},\bar{y^{g}}\geq 0,\theta \geq 0,\\ x\in R^{m},y^{g}\in R^{S1},y^{b}\in R^{S2} \end{array} \end{array}\right.\\ X=[x_{1},x_{2},\cdots ,x_{n}]\in R^{m\times n},Y^{g}=[y_{1}^{g},y_{2}^{g},\cdots ,y_{n}^{g}]\in R^{S_{1}\times n},Y^{b}=[y_{1}^{b},y_{2}^{b},\cdots ,y_{n}^{b}]\in R^{S_{2}\times n} \end{array}$$ (2)

Eq (2) is premised on the constant size assumption, where the *S* = (*S*^−^,*S*^*g*^,*S*^*b*^) vector represents the input, desired and undesired output slacks, respectively, and the ρ* objective function characterizes the efficiency of the decision-making unit. Input and output variables are initially determined, employing the approach introduced by Wu et al. [51]. Specifically, labor, capital stock, and energy are chosen as inputs, while GDP is designated as the desired output. Additionally, industrial sulfur dioxide, wastewater emissions, and general industrial solid particulate matter are identified as undesired outputs. The involved variables and relevant data descriptions are outlined in Table 2.

**Table 2:** Input-output index system of GTFP measurement.

Indicator category	name of index	Specific index selection and unit
Investment index	Labor input	Number of employees in each region
	Capital input	The capital stock of materials / 100 million yuan
	Energy input	Total energy consumption in each region / 10,000 tons of standard coal
Output indicators	Expect output	Actual GDP of the region / 100 million yuan
	Undesired output	The SO_2_ emission in the exhaust gas / ten thousand tons
		Waste water discharge capacity / ten thousand tons
		General industrial solid waste output / ten thousand tons

#### 4.3.2 Core explanatory variable

The PFTZs are analyzed in this paper. The 12 PFTZs cities established in the YREB from 2013 to the end of 2019 are selected as the treatment group, while the remaining 87 cities form the control group. Based on the announcement of the establishment time of PFTZs by the National Development and Reform Commission, the year of the establishment of PFTZs and the subsequent years of the treatment group are assigned as 1, and the other years are assigned as 0.

#### 4.3.3 Control variable

In order to mitigate the potential endogeneity of other variables on urban green development, this study opts for five control variables.

**Economic development** Economic development is a crucial determinant of GTFP growth, and this study utilizes the approach from Hao et al. [52] to quantify economic development (PGDP) by applying the natural logarithm of per capita gross regional product.

**Education developmen**t Educational development is acknowledged as a crucial factor in enhancing economic growth and productivity. In this study, the level of educational development (EDU) is quantified by calculating the logarithm of the general tertiary institution enrollment numbers.

**Financial development** In this paper, we cite Ren et al. [42] who define financial development level (DFIN) as the ratio of the year-end balance of loans from financial institutions to the gross regional product of the year.

**Trade development** Foreign direct investment has the potential to bring in advanced production technology and management theories, as well as facilitate the transfer of knowledge and experiences to enhance regional GTFP. This study references Cavallino [53] to quantify the extent of foreign trade (OPEN) using the ratio of actual foreign investment to regional GDP.

**Infrastructure construction** Infrastructure development (INFR) can significantly impact the efficiency of factor utilization and has a complex influence on GTFP. In this study, we utilize the comprehensive index measure developed by Zhang et al. [54], which includes transportation, communication, energy, environment, and healthcare, constructed using the entropy method.

#### 4.3.4 Mediating variables

**Technological Advancement** The typical Research and Development (R&D) staff are skilled professionals with a high level of expertise and creativity. The full-time equivalents of R&D staff serve as an indicator of an organization’s capability in technological innovation. This study utilizes Huang et al. [55] as a reference to quantify technological advancement (TECH) through the logarithm of R&D personnel’s full-time equivalents.

**Industrial upgrading** Industrial upgrading (IS) is often accompanied by a transformation of the economic structure, with the secondary sector mainly comprising manufacturing and construction, while the tertiary sector encompasses the service industry. When a country’s or region’s economy gradually shifts from being dominated by manufacturing to being dominated by services, the growth of the tertiary industry may outpace that of the secondary industry. Therefore, observing the ratio of the GDP of these two industries can reveal the evolution of the economic structure and the trend of industrial upgrading. As a result, the ratio of the gross domestic product of the secondary industry to that of the tertiary industry is utilized [56].

**Government intervention** The general budget expenditure of local finance is a reflection of the government’s fiscal expenditure at the local level. Through fiscal expenditure, the government provides public services, infrastructure construction, social welfare support, and more. Local fiscal general budget expenditure can be a primary method of government intervention in economic activities at the local level. This study draws on Chang et al. [3] and uses the ratio of local general fiscal expenditure to Gross Regional Product to indicate the level of government intervention (GOV).

## 5. Empirical analysis

### 5.1 Benchmark regression

In order to assess the impact of the establishment of PFTZs on urban green development, this study employs the multi-period DID method for empirical analysis (specific results are presented in Table 3), wherein the coefficient estimate of PFTZs signifies the average treatment effect pre- and post-establishment of PFTZs. As demonstrated in Table 3, the regression coefficients of the primary explanatory variables consistently exhibit significant positive values irrespective of the addition of control variables. Additionally, there exists a significant positive correlation between PFTZs establishment and GTFP, indicating that the establishment of PFTZs can facilitate the green development of cities in the YREB. Column (2) highlights that the construction of PFTZs in a city within the YREB region leads to a 9.88% enhancement in the quality of green development. H1 is confirmed.

**Table 3:** Baseline regression results.

	(1)	(2)
	GTFP	GTFP
PFTZ	0.1414***	0.0988***
	(9.5246)	(6.8140)
PGDP		0.0030
		(1.6065)
EDU		-0.0013*
		(-1.8257)
DFIN		0.0000***
		(7.1440)
OPEN		-0.0000***
		(-4.1141)
INFTA		0.0299***
		(4.7717)
Year FE	Yes	Yes
City FE	Yes	Yes
_cons	1.0337***	0.6695**
	(2100.8891)	(2.0921)
*N*	1485	1485
adj. *R*^2^	0.7161	0.7274

*t* statistics in parentheses

* *p <* 0.1

** *p* < 0.05

*** *p* < 0.01.

### 5.2 Parallel trend test

**Fig 2:**
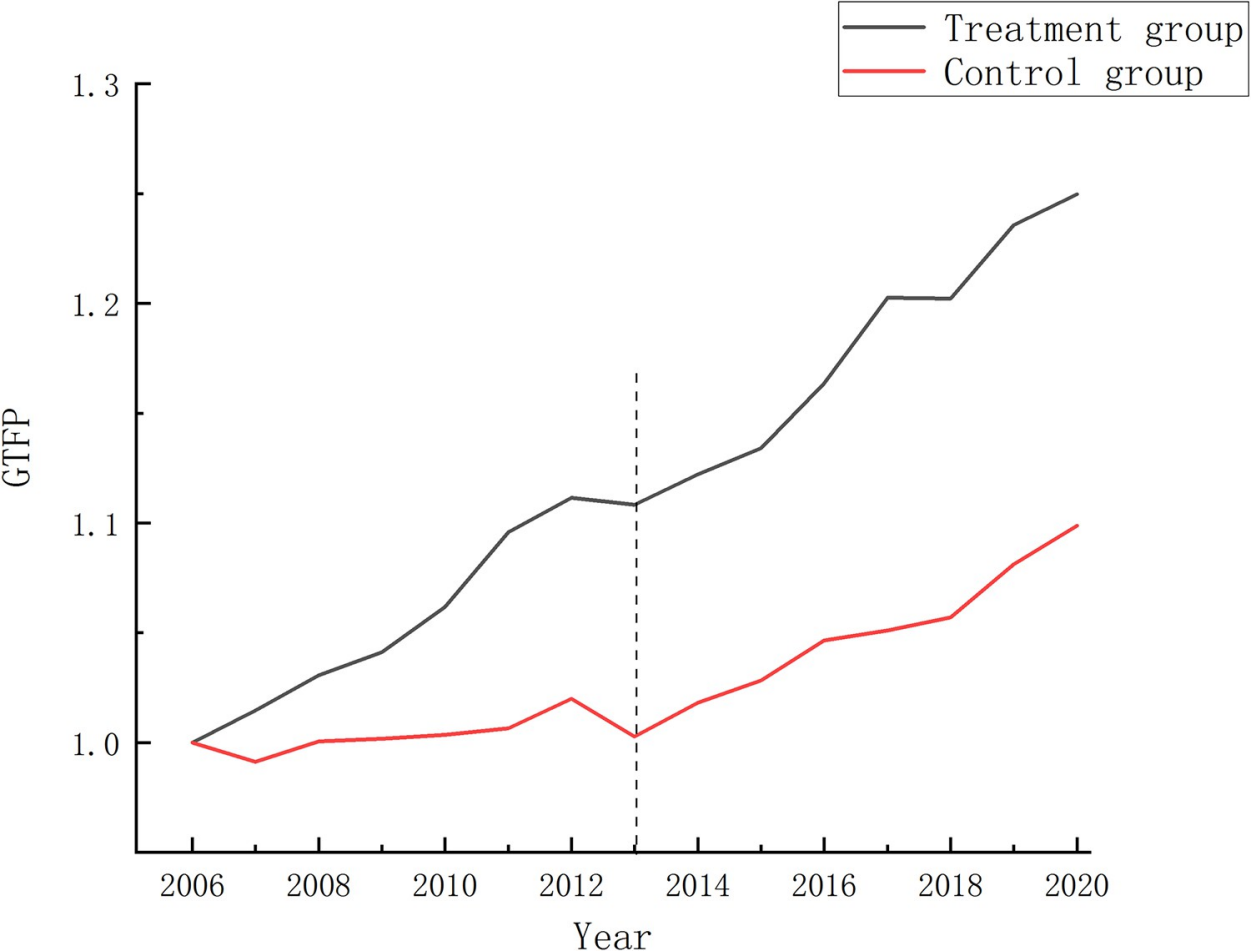
Trend of change in mean green total factor productivity for treatment and control groups. Trend of change in the mean value of GTFP for treatment and control groups.

The essential prerequisite for utilizing the double-difference method is that the treatment and control groups exhibit parallel development trends prior to policy implementation. This means there should be no significant disparity in the trend of GTFP changes between the treatment and control groups in the YREB region, regardless of whether a city establishes a PFTZ. This study employs the time trend graph and event study method to collectively assess the parallel trend. The time trend graph illustrates the mean GTFP of PFTZs pilot cities and non-pilot cities within the YREB region, as depicted in Fig 2. The analysis reveals that the change in the mean GTFP value for PFTZ pilot cities and non-pilot cities during 2006–2013 follows a similar pattern, confirming the parallel stable trend hypothesis. PFTZs, as focal points for openness and policy innovation, possess substantial autonomy and preferential policies that can drive urban green development.

Since the time trend graph can only be assessed intuitively, it does not differentiate between the treatment and control groups in a statistically significant manner. This study builds upon Beck [50] and others to develop a time-analytic model for conducting a parallel trend test. The specific model is as follows:

$$GTFP_{it}=\lambda_{0}+\sum_{d\geq -5,d\neq 0}^{5}\rho_{d}D_{it}^{d}+\vartheta_{j}Control_{it}+\varphi_{t}+\delta_{i}+\varepsilon_{it}$$ (3)

**Fig 3:**
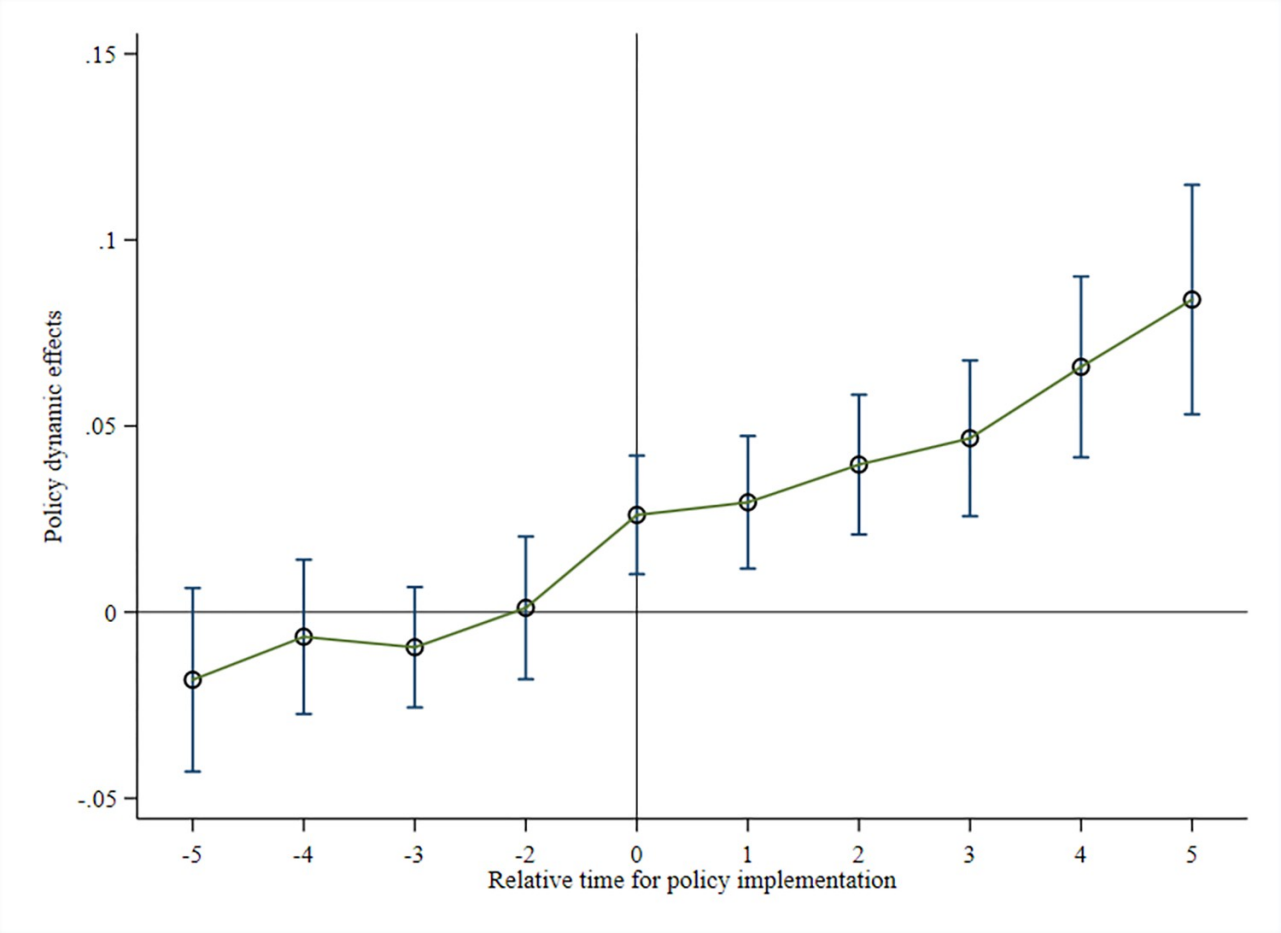
Parallel trend test chart.

The variable $D_{it}^{d}$ is a relative year policy variable generated by using the year of implementation of the PFTZ policy as the base period. $D_{it}^{d}$ is assigned to 1 if the city *i* piloted the PFTZ policy in *d*, and 0 otherwise. Where, variable $D_{it}^{d}$ serves as the policy dummy variable. If city *i* has implemented the PFTZ policy in year *d*, then $D_{it}^{d}$ is assigned the value 1; otherwise, it is assigned 0. $D_{it}^{d}=1$ when *d* = −5,−4,⋯,4,5, 0 otherwise. $D_{it}^{d}=1$ when *d*≥5, 0 otherwise. The significance of the parameter *ρ*_*d*_ and the trend of the change can verify that the model satisfies the parallel trend assumption, enabling further analysis of the temporal heterogeneity of PFTZs effect on GTFP (regression results are illustrated in Fig 3).

From the change in coefficient *ρ*_*d*_ over time in Fig 3 (with a 95% confidence interval), it can be inferred that the confidence intervals of coefficient from the first 5 years to the year before the establishment of PFTZs contain the horizontal line 0. This indicates that the treatment group and the control group exhibit the same trend of change before the establishment of PFTZs and satisfy the parallel trend assumption. From the 1st to the 5th year after the establishment of PFTZ, GTFP shows a significant and gradual upward trend, indicating a sustained effect of PFTZ on urban green development, with the effect strengthening over time.

### 5.3 Robustness tests

#### 5.3.1 Counterfactual tests

In order to mitigate potential time-related factors influencing the GTFP outcomes of the treatment and control group cities, this study, built on the counterfactual concept, sequentially hypothesizes the implementation of the PFTZ policy 1, 2, and 3 years prior to, and 1, 2, and 3 years following the present time. These are represented as PFTZ^pre1^, PFTZ^pre2^, PFTZ^pre3^, PFTZl^ag1^, PFTZ^lag2^, and PFTZ^lag3^, respectively. Dummy variables were then incorporated into the baseline regression model to conduct a placebo test at the time level. If the regression coefficients of the primary explanatory variables are significantly positive either prior to or with a lag of several years, it indicates that the PFTZ policy may not be the primary driving force behind the city’s green development enhancement and could potentially be influenced by other factors or policies. Typically, it is assumed that PFTZ requires one year from approval to policy implementation, and the regression coefficients of the default lag period also need to pass the counterfactual test if they are significant. The results revealed in Table 4, by removing PFTZ^lag1^, PFTZ^pre1^, PFTZ^pre2^, PFTZ^pre3^, PFTZ^lag2^, and PFTZ^lag3^ coefficient estimates, are not significant. This suggests that there is no systematic difference in the time trend between the treatment and control group cities, further demonstrating that the establishment of the PFTZs fosters the city’s development.

**Table 4:** Counterfactual test.

Explained variable: GTFP	(1)	(2)	(3)	(4)	(5)	(6)
	PFTZ^pre3^	PFTZ^pre2^	PFTZ^pre1^	PFTZ^lag1^	PFTZ^lag2^	PFTZ^lag3^
PFTZ*	0.0533	0.0745	0.0896	0.1490*	-0.0469	-0.0867
	(1.1291)	(1.4169)	(1.5765)	(1.7029)	(-0.4782)	(-0.7805)
*Control*	*Yes*	*Yes*	*Yes*	*Yes*	*Yes*	*Yes*
*City FE*	*Yes*	*Yes*	*Yes*	*Yes*	*Yes*	*Yes*
*Year FE*	*Yes*	*Yes*	*Yes*	*Yes*	*Yes*	*Yes*
_cons	1.4016**	1.3793**	1.3566**	1.2766**	1.4715**	1.4931**
	(2.3272)	(2.3276)	(2.3184)	(2.3166)	(2.1785)	(2.182)
*N*	1485	1485	1485	1485	1485	1485
adj. *R*^2^	0.2825	0.284	0.2851	0.2887	0.2813	0.2819

*t* statistics in parentheses

* *p <* 0.1

** *p* < 0.05

*** *p* < 0.01.

#### 5.3.2 PSM-DID test

The most optimal scenario when using the DID method is that the treatment group cities and control group cities are randomly chosen. Considering that the establishment of PFTZ is a strategic decision at the national level, its implementation requires comprehensive consideration of the city’s economic and social development level, degree of openness to the outside world, and geographic conditions before implementation. Therefore, there is subjectivity in the site selection process, treating the construction of the PFTZ as a quasi-natural experiment. This may lead to sample selectivity errors during the treatment and experimental group processing, impacting the experiment results. To mitigate the inherent disparity between these cities and reduce the impact of endogeneity in policy choice on the estimation results, we refer to Wang [27] and use control variables as covariates and the propensity score matching method, including radius matching, kernel matching, and near-neighbor matching. These three matching methods are applied yearly for the treatment group to identify similar cities and form a new sample. Based on the results of propensity score matching and double differencing as shown in Table 5, the regression coefficients of the core explanatory variables are significantly positive at 1% and 5% levels, respectively. These coefficients are not significantly different from the benchmark regression results, indicating the robustness of the conclusion that the establishment of PFTZs promotes the green development of cities.

**Table 5:** PSM-DID test.

Explained variable: GTFP	(1)	(2)	(3)
	Radius matching	Kernel matching	near-neighbor matching
PFTZ	0.0654***	0.0948***	0.0755***
	(2.7343)	(5.1591)	(12.0102)
*Control*	Yes	Yes	Yes
_cons	0.3431***	0.6081***	0.7049***
	(4.9048)	(24.2156)	(12.2657)
*N*	553	260	446
adj. *R*^2^	0.2258	0.2157	0.8339

*t* statistics in parentheses

* *p <* 0.1

** *p* < 0.05

*** *p* < 0.01.

## 6. Extensibility analysis

### 6.1 Analysis of impact mechanisms

The previous benchmark regression results and a series of robustness tests have confirmed that the establishment of PFTZs can facilitate urban green development. The mechanism through which this effect is achieved is outlined based on previous theoretical analysis, indicating that PFTZs primarily drives urban green development by accelerating technological progress, promoting industrial upgrading, and reducing government intervention, among other channels. In this regard, this paper refers to the existing literature to set up the following model for empirical testing [3,57].

$$M_{it}=\lambda_{0}+\lambda_{1}PFTZ_{it}+\lambda_{2}Control_{it}+\varphi_{t}+\delta_{i}+\varepsilon_{it}$$ (4)

$$GTFP_{it}=\gamma_{0}+\gamma_{1}PFTZ_{it}+\gamma_{2}M_{it}+\gamma_{3}Control_{it}+\varphi_{t}+\delta_{i}+\varepsilon_{it}$$ (5)

when *M*_*it*_ represents the mediating variables denoting technological innovation, industrial upgrading, and the degree of government intervention, respectively. *λ*_1_ indicates the impact of PFTZs on the mediating variable, while *γ*_2_ indicates the impact of the mediating variable on GTFP. If both *λ*_1_ and *γ*_2_ are statistically significant, it demonstrates the existence of the influence mechanism. If at least one of them is significant, conducting the Sobel test becomes necessary. If the p-value is less than 0.1 after the test, it indicates the existence of the influence mechanism. The regression findings are displayed in Table 6.

**Table 6:** Mechanisms of the impact of the PFTZ on the GTFP in cities.

	(1)	(2)	(3)	(4)	(5)	(6)	(7)
	GTFP	TECH	GTFP	IS	GTFP	GOV	GTFP
PTFZ	0.0988***	2.8887**	0.0769***	0.3642***	0.0756***	-0.0211**	0.0838***
	(6.8140)	(2.4736)	(4.7963)	(3.8438)	(4.8395)	(-2.1375)	(6.0599)
TECH			0.0052**				
			(2.0840)				
IS					0.0637**		
					(3.0911)		
GOV							-0.3291***
							(-4.0319)
_cons	0.6695**	1.9918***	0.9379***	-0.1124	0.7355***	0.2576**	0.4957***
	(2.0921)	(5.0855)	(44.1294)	(-0.4789)	(12.7290)	(2.1849)	(3.9984)
Control	Yes	Yes	Yes	Yes	Yes	Yes	Yes
*City FE*	Yes	Yes	Yes	Yes	Yes	Yes	Yes
*Year FE*	Yes	Yes	Yes	Yes	Yes	Yes	Yes
*N*	1485	1485	1485	1485	1485	1485	1485
adj. *R*^2^	0.7274	0.9337	0.7264	0.8355	0.7264	0.7610	0.6922

*t* statistics in parentheses

* *p <* 0.1

** *p* < 0.05

*** *p* < 0.01.

In Table 6, it is demonstrated that the core variable PFTZ has a significant positive impact on the variable GTFP in column (1). Columns (2) and (3) display the regression results related to the mechanism of technological progress. Column (2) indicates that PFTZ has a significant positive effect on the mediator variable TECH, suggesting that the establishment of PFTZ can notably enhance technological progress. From column (3), it can be observed that technological progress significantly promotes GTFP. Combining columns (2) and (3), it is evident that the coefficients of PFTZ remain consistent in terms of direction and significance even after adding the mediating variable, although the magnitude of the effect is reduced. This result implies that the establishment of PFTZ can stimulate green technological progress and effectively promote urban green development. Columns (4) and (5) present the regression results concerning the mechanism of industrial upgrading. Similarly, the coefficients of PFTZ remain unchanged when mediating variables are added, albeit with a reduced effect size. This suggests that PFTZ can significantly foster urban industrial upgrading. Columns (6) and (7) focus on the mechanism of government intervention. Government intervention and GTFP exhibit a notable negative correlation, and after adding the mediating variable, the coefficients of PFTZ remain unchanged, though the effect size increases. This implies that the establishment of PFTZ can optimize resource allocation and enhance the process of urban green development by reducing government intervention. Through the analysis of mediating effects, all three mechanisms through which PFTZ promotes urban green development are validated, confirming H2.

### 6.2 Heterogeneity analysis

#### 6.2.1 Batch heterogeneity

Referring to existing studies [58], Eq (6) is set on the basis of Eq (1) in order to examine the impact of different batches of PFTZs established in the YREB region on the green development of the city.


$$GTFP_{it}=\alpha_{0}+\sum_{j=1}^{3}\alpha_{j}PFTZ_{it}\times Batch_{jit}+\alpha_{2}Control_{it}+\varphi_{t}+\delta_{i}+\varepsilon_{it}$$ (6)

During the sampling period in this study, three batches of PFTZs were established, denoted as *Batch*_*jit*_, *j* = 1,2,3. If the city belongs to the first batch of PFTZ establishment, denoted as *Batch*_1*it*_ = 1, and so on until reaching *Batch*_2*it*_, *Batch*_3*it*_. The heterogeneity of the impact of different PFTZ establishment batches on urban green development can be evaluated by comparing the absolute size of the estimated PFTZ coefficients for each batch.

The regression results are displayed in the first column of Table 7. It is evident that the impacts of the initial and second waves of PFTZ establishment on urban green development are largely similar, while the third wave has a comparatively weaker promotional effect. This could be attributed to the fact that the first two waves of PFTZ were established relatively early, and the policy implementation was relatively robust, enabling the PFTZ to actively drive technological progress, optimize industrial structural upgrades, and enhance resource utilization efficiency, thus significantly contributing to the city’s green development. In contrast, the third wave of PFTZ was established in 2019, and its policy implementation is still in the early stages, thus not yet fully maximizing its potential in promoting the city’s green development.

**Table 7:** Heterogeneity analysis.

	(1)	(2)
	Batch heterogeneity	Regional heterogeneity
First PFTZ	0.0755***	
	(5.5888)	
Second PFTZ	0.0740***	
	(4.0268)	
Third PFTZ	0.0485**	
	(2.4566)	
Upper		0.0922***
		(6.3163)
Middle		0.0748***
		(2.4580)
Lower		0.0431***
		(2.8146)
Control	Yes	Yes
City FE	Yes	Yes
Year FE	Yes	Yes
_cons	0.6643*	0.7007**
	(2.0459)	(2.1946)
N	1485	1485
adj. R2	0.7272	0.7276

*t* statistics in parentheses

* *p <* 0.1

** *p* < 0.05

*** *p* < 0.01.

#### 6.2.2 Regional heterogeneity

The YREB spans across eastern, central, and western China. It is divided into the upper, middle, and lower reaches of the Yangtze River based on its location, and exhibits significant heterogeneities in terms of economic development, geographic location, resource endowment, and institutional policies. These heterogeneities are analyzed with reference to Delgado and Florax [59] to construct a regional heterogeneity model for analysis.

The regression findings are presented in column (2) of Table 7. It is evident that the establishment of PFTZs in the YREB region has notably contributed to the eco-friendly development of cities. However, the upstream and middle reaches of the Yangtze River exhibit significantly stronger impact compared to the lower reaches. This could be attributed to various factors. Firstly, cities in the lower reaches tend to have larger populations and city sizes, resulting in more resource consumption and environmental pressure. This requires more intricate policy adjustments to promote green development within these large urban systems. Secondly, the lower reaches usually have more developed manufacturing and trading industries, leading to a higher reliance on traditional high-pollution industries. Restructuring the economies of these cities will therefore take time.

### 6.3 Examination of spatial spillover impacts

The preceding analysis validates the promotional impact of PFTZs construction on urban green development. However, the spatial correlation between districts is frequently disregarded during the empirical process, leading to potential bias in the estimation results. Is there evidence of a spatial spillover effect? If so, what is the magnitude of this effect? This study draws upon the research methodology of Paliska [60] and incorporates the spatial econometric model (7) for further exploration.


$$GTFP_{it}=\rho (W_{it}⊗\xi ')GTFP_{it}+\lambda_{1}PFTZit+\lambda_{2}Control_{it}+\varphi_{t}+\delta_{i}+\varepsilon_{it}$$ (7)

In this case, when *k* = 1 happens, *W*_1_ represents a spatial matrix developed based on geographical distances between cities Although the geographical distance weight can to some extent reflect the relationship between cities, the green development of cities with close geographical distances is not necessarily relevant. In order to explore whether there is a spatial spillover effect in economically adjacent areas, this article constructs an economic distance spatial matrix, that is, when *k* = 2 is the economic distance spatial weight matrix *W*_2_. Using the average of the city GDP after reducing it as the base year in 2006, the calculation is as follows:

Wijc=1|GDPi−GDPj| (8)

where, *GDP*_*i*_ is the average of the deflated city GDP for the 2006 base period.

#### 6.3.1 Global spatial correlation test

**Fig 4:**
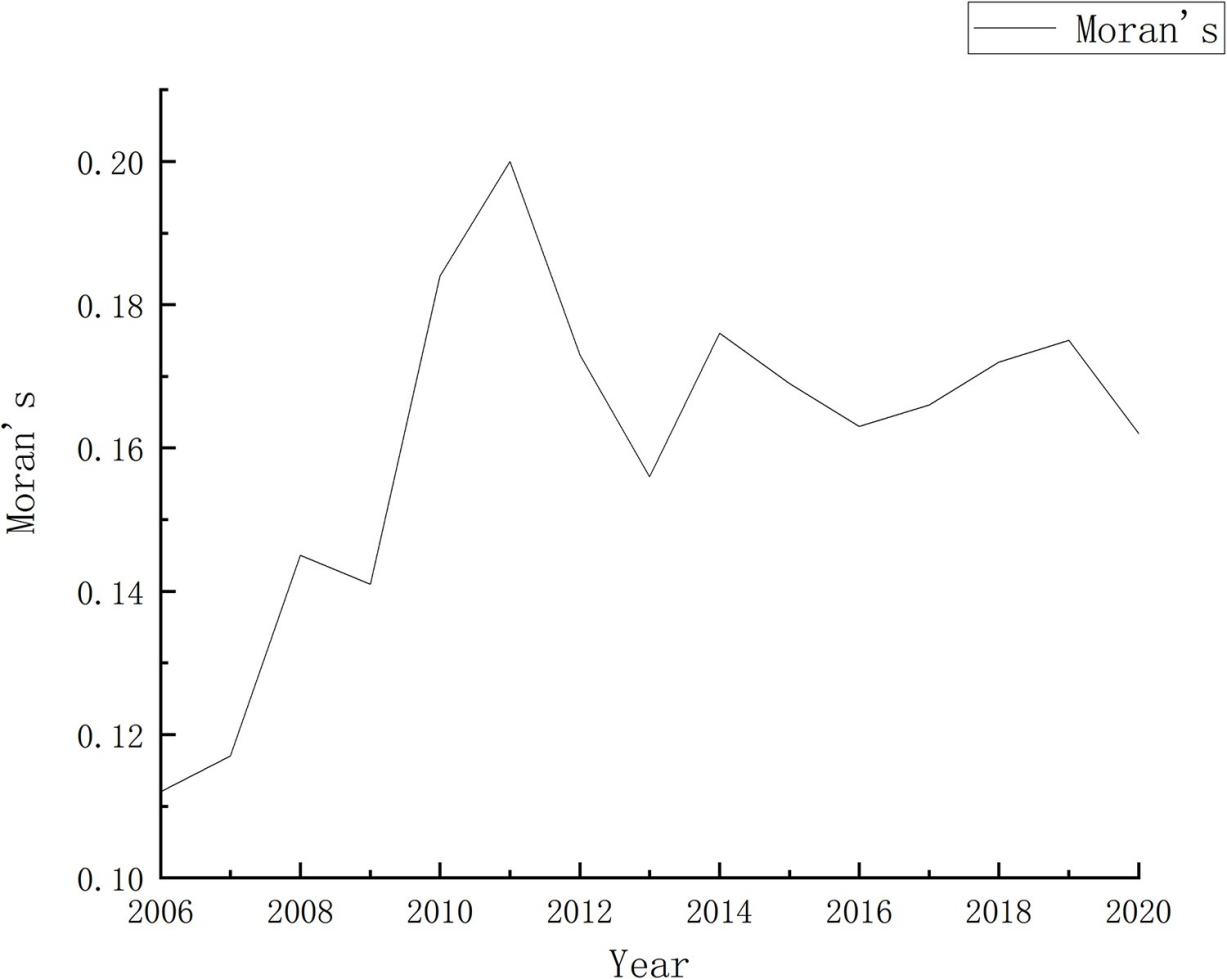
Distribution of the Moran Index.

The important premise for spatial regression is spatial autocorrelation. The results of the Moran’s I test conducted in this study are shown in Fig 4. It can be seen that the Moran’s I values are all greater than 0, and the p-values are all less than 0.05, which preliminarily indicates the existence of spatial autocorrelation.

#### 6.3.2 Spatial spillover regression results

Table 8 presents the results of double difference spatial regression estimation, focusing on the robust empirical impact of the pilot policy on urban green development. The table specifically reports the regression with GTFP as the explanatory variable under different spatial matrices. Columns (1) and (2) of Table 8 show that the spatial regression coefficient ρ is statistically significant at the 1% level under both the distance matrix and the economic matrix, indicating a substantial spatial dependence in the city’s green development. This implies that the green development of spatially connected cities has a significant spatial spillover effect on each other. Moreover, the indirect effect of the PFTZ policy reveals a significant spatial spillover effect, amplifying the total impact on the city’s green development due to spatial relevance. These findings support the effectiveness of the pilot policy in enhancing the city’s green development. Combined with empirical studies, hypothesis 3 was validated.

**Table 8:** Decomposition of spatial spillover effects.

	(1)	(2)
	W_1_	W_2_
LR_Direct	0.0727***	0.0607***
	(5.7947)	(1.8918)
LR_Indirec	0.0789**	0.0702***
	(1.9696)	(5.6692)
LR_Total	0.1515***	0.1309***
	(3.2372)	(3.3582)
ρ	0.5015***	0.4431***
	(5.3537)	(4.4279)
Log-L	1967.0911	1991.4084
R^2^	0.2126	0.2748

## 7. Conclusions and research recommendations

Achieving high-quality economic development necessitates a balanced approach to the relationship between the economy and the environment. As a major national strategy for opening up in China’s new era, the construction of PFTZs should not only focus on economic benefits and reform, but also prioritize green development. Given this context, based on theoretical analysis, this paper employs panel data from cities within the PFTZs along the YREB from 2006 to 2020 to assess the impact of PFTZs construction on urban green development. The study finds that the establishment of PFTZs has played a positive role in promoting green development in cities along the YREB. Moreover, differences in the timing and location of the establishment of these zones have resulted in varying degrees of impact. These conclusions have been supported by various robustness tests. Further empirical research based on transmission mechanisms reveals that the construction of PFTZs can drive urban technological progress, optimize industrial structures, promote industrial upgrading, and accelerate the green development process in the YREB. The implementation of policies such as the negative list management model in PFTZs have reduced government intervention, increased marketization, improved resource allocation efficiency, and further promoted urban green development. Spatial analysis also shows that the policies implemented in PFTZs not only have a significant impact on the green development of the cities where they are located, but also drive the green development of neighboring cities. By focusing on the PFTZs along the YREB, this paper not only confirms the positive role of PFTZs construction in promoting urban green development, deepening our understanding of the relationship between PFTZs construction and urban green development, but also provides important insights for exploring green development paths from the perspective of promoting PFTZs construction.

Based on the conclusions of this paper, the construction of PFTZs have initially demonstrated its promotional effects on the green development of cities along the YREB. However, it is noteworthy that there are still disparities in the policy effects of PFTZs located in the upstream, midstream, and downstream regions of the Yangtze River. Irresistible factors such as geographical location, resource endowments, and the foundation for green development can all influence the effectiveness of policy implementation. Therefore, during the construction of PFTZs, it is crucial to adhere to the principle of adapting measures to local conditions. Full consideration should be given to the differences in implementing pilot policies among the upstream, midstream, and downstream regions of the Yangtze River. Differential approaches should be explored to leverage the unique development characteristics and resource endowments of each city. By combining these with local development realities, the generalization of singular policies should be avoided. Instead, timely adjustments should be made to the direction and content of institutional innovations in PFTZs, fostering an innovative system and development pattern with local characteristics that promote urban green development. Moreover, the construction of PFTZs should guide technological innovation activities to focus on future technological iterations, driving industrial green upgrades. PFTZs can enhance the efficiency of urban green development by elevating the level of urban innovation [16] (Zhang et al., 2023). The government should fully leverage its role as a service-oriented government, paying attention to the carbon emission reduction effects brought about by technological progress. It should guide PFTZs to rationally utilize the technological spillover effects of foreign direct investment, improve resource utilization efficiency, and enhance the quality of urban green development.

The application of spatial econometric models to the implementation of policies in PFTZs reveals that in the process of institutional reform, reform measures can have positive or negative impacts on surrounding regions. This study finds that within the YREB, the implementation of PFTZs policies also has a positive effect on the green development of neighboring cities. Therefore, in the future process of implementing the “PFTZs Enhancement Strategy,” it is crucial to plan and layout ahead, improve the linkage development mechanism among PFTZs in the YREB, and promote coordinated regional development. Individual PFTZs should further simplify registration procedures, streamline approval processes, optimize customs clearance procedures, strengthen information and communication infrastructure construction, and promote talent mobility and training. This will facilitate the free flow and exchange of resources, information, and talent within and outside the region. At the same time, enhancing cooperation and linkage among enterprises in the upstream, midstream, and downstream regions of the Yangtze River, and jointly building collaborative development models such as “industry chain + value chain” and “industry chain + supply chain” will promote the sustainable economic development of the YREB. By leveraging the positive spillover effects of PFTZs policies, we can promote balanced and coordinated development across the entire region, fostering a green and sustainable economic landscape that benefits all cities along the Yangtze River. This approach aligns with the overall strategy of promoting high-quality development and building an open economy with higher standards.

## 8 Research limitations and directions

This paper has solely investigated the influence of the early PFTZs in the Yangtze River Delta region on the ecological development of the cities, and has not extensively analyzed the specific impact of each individual PFTZ in the YREB region on the ecological development of the cities where they are situated. Subsequent research will comprehensively examine the ecological economic impacts of each PFTZ in the YREB region.

## Supporting information

S1 Fig Mechanistic pathways for PFTZs to Influence Urban Green Development. (PDF)

S2 Fig Trend of change in mean green total factor productivity for treatment and control groups. (XLS)

S3 Fig Parallel trend test chart. (XLSX)

S4 Fig Distribution of the Moran Index. (XLSX)

S1 Table Planning of PFTZs within the YREB. (XLS)

S2 Table Input-output index system of GTFP measurement. (XLS)

S3 Table Baseline regression results. (XLSX)

S4 Table Counterfactual test. (XLS)

S5 Table PSM-DID test. (XLSX)

S6 Table Mechanisms of the impact of the PFTZ on the GTFP in cities. (XLS)

S7 Table Heterogeneity analysis. (XLSX)

S8 Table Decomposition of spatial spillover effects. (XLSX)
