# Supplementary figures and images for: Research on the impact of pilot free trade zones on urban green development: A case study based on the Yangtze River Economic Belt in China
Source: PLoS One. 2024 May 24;19(5):e0303626. doi: 10.1371/journal.pone.0303626 (PMC11125538; doi:10.1371/journal.pone.0303626)

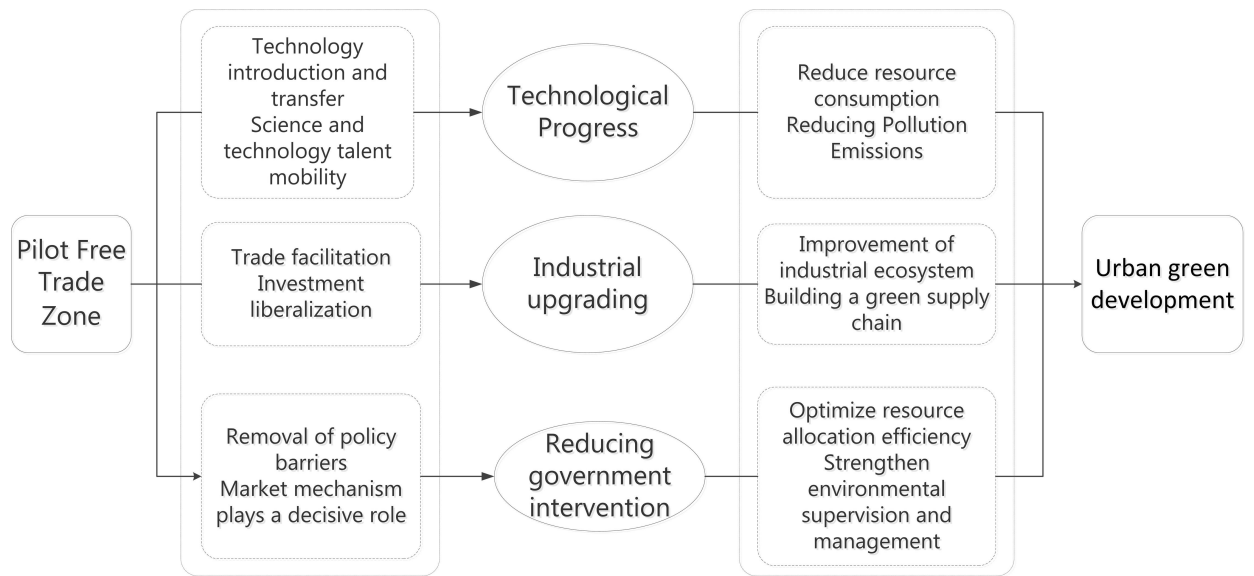

Supplement: S1 Fig — (PDF) [file pone.0303626.s001.pdf]
